# Emotional response in schizophrenia to the “36 questions that lead to love”: Predicted and experienced emotions regarding a live social interaction

**DOI:** 10.1371/journal.pone.0212069

**Published:** 2019-02-27

**Authors:** Elizabeth A. Martin, Mayan K. Castro, Lilian Y. Li, Emily J. Urban, Melody M. Moore

**Affiliations:** 1 Department of Psychological Science, University of California, Irvine, Irvine, CA, United States of America; 2 Center for Healthy Minds, University of Wisconsin-Madison, Madison, WI, United States of America; Icahn School of Medicine at Mount Sinai Hospital, UNITED STATES

## Abstract

Evidence suggests that individuals with schizophrenia (SZ) report anticipatory pleasure deficits compared to controls and that these deficits are linked to decreased motivation to engage socially. However, these deficits have been identified via self-report measures of hypothetical pleasant stimuli, leaving it unclear whether they exist in reference to actual social situations. To address this issue, we created a live social interaction that minimized the reliance of higher-order cognitive processes. SZ and control participants were told that they would be playing an "enjoyable sharing game" with another study participant (who was actually a confederate) that involved asking and answering questions (36 interpersonal closeness generation questions; Aron et al., 1997). Participants then reported their current mood and the emotions they anticipated experiencing during the pleasant social interaction. Immediately following the interaction, they reported their experienced emotions. We found that the SZ group anticipated more negative emotion (*d* = 1.0), but were less accurate in forecasting negative emotion (*d* = .81), than controls, and these effects were large. There were small, non-significant group differences in anticipation, experience, and accuracy in forecasting of positive emotion (all *d*s < .29). Also, social anhedonia was positively correlated with anticipated negative affect and negatively associated with experienced positive emotion. At the same time, controls reported finding the interaction to be a more positive emotional experience overall, *d* = 0.75. This is the first study to show that "anticipatory pleasure deficits" in SZ might actually be heightened anticipated negative emotion and that inaccurate forecasting could be linked to decreased social motivation.

## Introduction

Schizophrenia is associated with impairments in emotion [1–3]. For example, many people with schizophrenia have elevated anhedonia [4]. Social anhedonia refers to reports of diminished experience of positive emotion in response to social stimuli [4,5]. Social anhedonia predicts future onset of the disorder [6,7], and in people with the disorder, it is associated with decreased social motivation and is currently not well treated [8]. Hence, understanding the nature of emotion processing deficits related to anhedonia in schizophrenia could potentially help prevent the disorder as well as treat functional disability in people with the disorder. However, the nature of emotion processing mechanisms in anhedonia are still unclear [9], including differences related to affective forecasting, or the ability to forecast one’s feelings regarding a future event [10]. Given the impact of affective forecasting on motivation to engage in goal-directed behavior [11,12], the current study examined the relationship between predicted and experienced emotion in schizophrenia in response to a live social interaction that has been shown to create feelings of closeness and intimacy between strangers [13].

In the schizophrenia literature, affective forecasting is almost exclusively considered in terms of anticipatory pleasure, defined as the “experience of pleasure related to future activities” ([14], p. 254), and are often contrasted with reports of consummatory pleasure, or pleasure experienced “in-the-moment.” Findings have been mixed in schizophrenia with some reporting reductions in anticipatory, but not consummatory, pleasure [14], others reporting reductions of consummatory, but not anticipatory, pleasure [15], and still others report deficits in both [16]. One possible explanation for these mixed findings is the reliance of self-report measures that involve queries about hypothetical events. That is, because of the associated cognitive deficits in schizophrenia (e.g., abstract thinking, memory) [17], some individuals may have more of a difficulty predicting how they might feel regarding an imaginary event than others. Thus, utilizing a live social situation in the laboratory minimizes the reliance on longer-term memory processes and abstract thinking, because emotional assessments can be taken immediately before and after the experimentally-controlled social event.

Similar to positive emotion, there is evidence that there are differences in negative emotion between individuals with and without schizophrenia, and these differences are related to anhedonia [9,18]. In a meta-analysis, Cohen and Minor [1] reported that group differences in mean negative ratings in response to positive stimuli were large in magnitude (Hedges *D* = .72). At the same time, clinician-rated anhedonia is associated with increased state and trait negative emotion [19,20]. In addition, using a data-driven approach, Strauss and Herbener [20] identified a specific cluster of individuals with schizophrenia who found negative stimuli more unpleasant, but didn’t differ in responses to positive stimuli, compared to controls, and this cluster was characterized by significantly higher levels of anhedonia. However, in contrast to the literature on forecasts of positive emotion, there is a dearth of investigations on forecasting of negative affect in schizophrenia. A single daily diary study that collected forecasts of negative emotion found that individuals with schizophrenia predicted more negative emotion than they reported experiencing over the course of a week [21]. Although interesting, no associations with anhedonia or comparisons with a control group were reported, making it unclear to what extent anhedonia is associated with forecasts of negative emotion, or whether accuracy in predictions of negative emotion differs between groups. Given that predictions of future negative and positive emotion influence motivation [11], the current study examined forecasts of both as well as the accuracy of these predictions.

The current study was the first to create a live social interaction for individuals with schizophrenia that was designed to be pleasant and create feelings of closeness and intimacy between members of the dyad meeting for the first time. We used the Interpersonal Closeness Generation task [13], which has been touted by the New York Times as “36 questions that lead to love” [22]. If the schizophrenia group has anticipatory pleasure deficits, we would expect them to report anticipating less positive emotion than control participants. If they have consummatory pleasure deficits, we would expect them to report experiencing less positive emotion than control participants. If anhedonia is related to affective forecasting, we would expect there to be significant relationships between self-reported anhedonia and predicted positive/negative emotion. At the same time, if anhedonia is related to the experience of in-the-moment emotion, we would expect there to be significant relationships between anhedonia and experienced emotion. Last, if schizophrenia is associated with less accurate predictions of emotion, we expect there to be group differences in accuracy.

## Materials and methods

### Participants

The schizophrenia (SZ) group was comprised of 16 stable outpatients (i.e., no major changes in psychiatric medication in the last 3 months, no hospitalizations for psychiatric reasons for the last 6 months) recruited through the Veterans Affairs Hospital (*n* = 12) or community advertisements or Craigslist (*n* = 4). All had a *Diagnostic and Statistical Manual of Mental Disorders* (5^th^ ed.) diagnosis of schizophrenia (*n* = 8) or schizoaffective disorder (*n* = 8) based on the Structured Clinical Interview for *DSM–5* (SCID-5) [23]. Control participants were 30 individuals recruited through community advertisements and Craigslist. General exclusionary criteria included diagnosis of a substance abuse disorder within the past 6 months, diagnosis of mental retardation, non-fluent English speakers, or a history of any neurologic event or disease (e.g., loss of consciousness for more than 10 minutes, stroke). In addition, control participants did not have any psychiatric diagnoses based on the SCID-5, and they denied having a first-degree relative with a psychotic disorder. Table 1 contains demographic and clinical information. The groups did not differ in age, sex, or race/ethnicity, all *p*s ≥ .1.

**Table 1:** Means (SD) of demographic and clinical measures by group.

	Schizophrenia Group (*n* = 16)	Control Group (*n* = 30)	Statistics
Age (years)	49.31 (13.33)	42.30 (12.95)	*t*(44) = 1.73, *p* = .1, *d* = .52, 95% CI [-.49, 1.51]
Sex (% female)	43.75%	60%	Fisher's Exact Test, *p* = .13
Race/ethnicity (%)			Fisher's Exact Test, *p* = .1
Caucasian, non- Hispanic	43.75%	46.67%	
African-American/ Black	37.5%	6.67%	
Hispanic	12.5%	20.0%	
Mixed race/other	6.25%	26.67%	
Brief Social Anhedonia Scale	6.06 (3.62)	2.0 (2.13)	*t*(42) = 4.70, *p* < .001, *d* = 1.45, 95% CI [.32, 2.55]
SANS Global Ratings			
Affective Flattening	0.94 (1.30)		
Avolition/Apathy	0.13 (0.48)		
Anhedonia-Asociality	1.31 (1.57)		
Attention	0.88 (0.99)		
SAPS Global Ratings			
Hallucinations	1.38 (1.87)		
Delusions	2.19 (1.70)		
Bizarre Behavior	0.13 (0.48)		
Positive Formal Thought Disorder	0.63 (1.11)		
CPZ equivalence	548.62 (334.36)		

Note. Do to a computer error, anhedonia measures were not collected for two individuals in the Control group.

### Materials

#### Social interaction task

The social interaction task was based on the Interpersonal Closeness Generation task (ICG) [13]. The ICG was first used in college undergraduate students to study self-disclosure and relationship building among randomly paired strangers. At the start of the task, participants were told they were going to participate in an "enjoyable sharing game" with another study participant who was going through the same study procedures as the participant when, in fact, the other study participant was one of two study confederates (for the full cover story provided to participant, see the Appendix in S1 File). No additional information was given to the participant (e.g., whether or not clinical status information was shared with the confederate), and no participant queried the experimenter for details about the confederate in the moments before the confederate was brought into the room. Hence, it was implied that the experimenter working with the participant had yet to meet the confederate because the confederate was undergoing the same research procedures with another experimenter. The confederates were both women with light skin, dark eyes, dark hair, and similar build. One was 31 years old and the other was 36 years old (mean age = 33.5 years). They did not significantly differ in age from either the schizophrenia group, *t*(16) = 1.63, *p* = .12, or control group, *t*(30) = 0.95, *p* = .35. They were blind to participant group status.

With chairs positioned approximately 2’ apart, the participant and confederate sat directly across from each other with nothing in between them (e.g., no table). Each member of the dyad asked and answered three sets of 10 questions designed to elicit feelings of closeness (e.g., "How is your relationship with your mother?"; "Do you have a hunch about how you might die?"). Specifically, as the series of questions progressed through the task, the content elicited via answers to the questions was supposed to be increasingly more intimate and personal. Participants were told to go through the questions in order and to take turns reading them aloud. They were also instructed to both answer each question before moving onto the next question. In addition, they were told it was not important to finish all of the questions in each set but rather they should take their time with each question, answering it thoroughly and thoughtfully. Last, participants were told the experimenter would tell them when it was time to move onto the next set of questions. To create as similar a social situation as possible for each participant, confederates gave the same answer to each question for every participant. The task lasted approximately 35 minutes. For questions and confederate responses, see the Appendix in S2 File.

#### Baseline emotion assessment

To assess participants' mood states before beginning the Social Interaction task, they were asked to indicate how much they were feeling four positive (happy, excited, relaxed, calm) and four negative emotions (sad, nervous, upset, fatigued) on a 1–5 scale (1 = Not at all, 5 = Extremely, both Cronbach’s α > .63).

#### Anticipatory emotion assessment

To assess how participants anticipated they would feel during the Social Interaction task, they were asked to indicate how much they thought they would feel the same eight emotions that were assessed at baseline immediately before the task began (both Cronbach’s α > .66). Due to a computer error, this assessment was not available for one control participant.

#### Experienced emotion assessment

Immediately following the Social Interaction task, participants were asked to indicate how they felt during the task using the same emotions items included in the baseline and anticipatory emotion assessments (both Cronbach’s α > .74). Due to a computer error, this assessment was not available for one control participant.

#### Interaction evaluation

Finally, participants completed an interaction evaluation [24]. This evaluation was comprised of 12 items from four subscales (all Cronbach’s α > .63): Emotional experience (e.g., How much did you enjoy the sharing game?), Engagement (How much do you feel you influenced the interaction?), Quality (e.g., How much did you consider the interaction to be smooth and natural?), and Disclosure (e.g., How much did you feel you disclosed to your game partner?). Participants were asked to indicate the extent to which they agreed with each item on a 1–8 scale (1 = Not at all, 8 = Very much).

#### Anhedonia measure

Participants completed the brief, 15-item version [25] of the Revised Social Anhedonia Scale (Cronbach’s α in the current study = .83) [26], which is designed to measure lack of relationships and lack of pleasure from relationships (e.g., “Having close friends is not as important as many people say”). It uses a true-false response format. Previous research has shown that the brief version of the Revised Social Anhedonia Scale has superior psychometric properties, particularly when used with an ethnically-diverse sample [27], as in the current study.

#### Clinician rated symptoms

All participants’ clinical symptoms were rated using the Scale for the Assessment of Negative Symptoms (SANS) [28] and Scale for the Assessment of Positive Symptoms (SAPS) [29]. These ratings were all completed by a PhD-level clinical psychologist (EAM), who has over 15 years of research and clinical experience working with this population. Based on the work of Strauss and colleagues [30] and Kotov and colleagues [31], we included negative symptom ratings captured with the Diminished Expression/Inexpressivity factor (sum score of select items from the Affective Flattening and Alogia SANS subscales) and the Avolition-Apathy factor (sum score of select items from the Avolition and Anhedonia-Asociality SANS subscales). Also, based on Kotov and colleagues [31], we included positive symptom ratings captured using the Reality Distortion factor (sum score of select items from the Hallucinations and Delusions SAPS subscales) and the Disorganization factor (sum score of select items from the Formal Thought Disorder and Bizarre Behavior SAPS subscales).

### Procedure

This study, which was approved by the Institutional Review Boards at both the University of California, Irvine (#2016–2945) and the Long Beach Veteran Affairs Hospital (#1436), was carried out in accordance with the latest version of the Declaration of Helsinki. All participants provided written informed consent to demonstrate capacity to consent.

After the clinical interview, all participants were fitted with an EEG cap. Participant, but not confederate, EEG data were collected. Those data are not reported in this manuscript.

Participants then completed the baseline emotion assessment. Next, the social interaction task was explained by an experimenter, and then participants completed the anticipatory emotion assessment. Then, the confederate, who was also wearing an EEG cap, was brought into the experiment room and connected to the amplifier. This was done to increase the believability of the cover story. Next, the participant and confederate completed the social interaction task. After, the confederate was escorted from the room, and the participant completed the experienced emotion assessment and interaction evaluation. Then, after a built-in break lasting approximately 30 minutes, the participant completed the interaction evaluation and the anhedonia questionnaire.

### Statistical plan

All statistical analyses were conducted using R [32]. Specific packages used to run HLM models in R included lme4 [33] and lmerTest [34]. To test whether there were group differences in emotion ratings as a function of time, we ran a mixed model of Group (Control, SZ) X Emotion (Positive, Negative) X Time (Baseline, Anticipated, Experienced). The model included random intercepts of subject. Significant interactions were followed-up with *t*-tests to aid in interpretation. To assess accuracy in affective forecasting, participants’ anticipated positive and negative emotion score were subtracted from their experienced positive and negative emotion scores, and *t*-test were conducted. Last, we tested for the relationships between self-reported social anhedonia and clinician-rated Anhedonia-Asociality with predicted and experienced emotional assessment scores and interaction evaluation scores.

## Results

### More anticipated and less accurate forecasting of negative emotion for the SZ group

**Fig 1:**
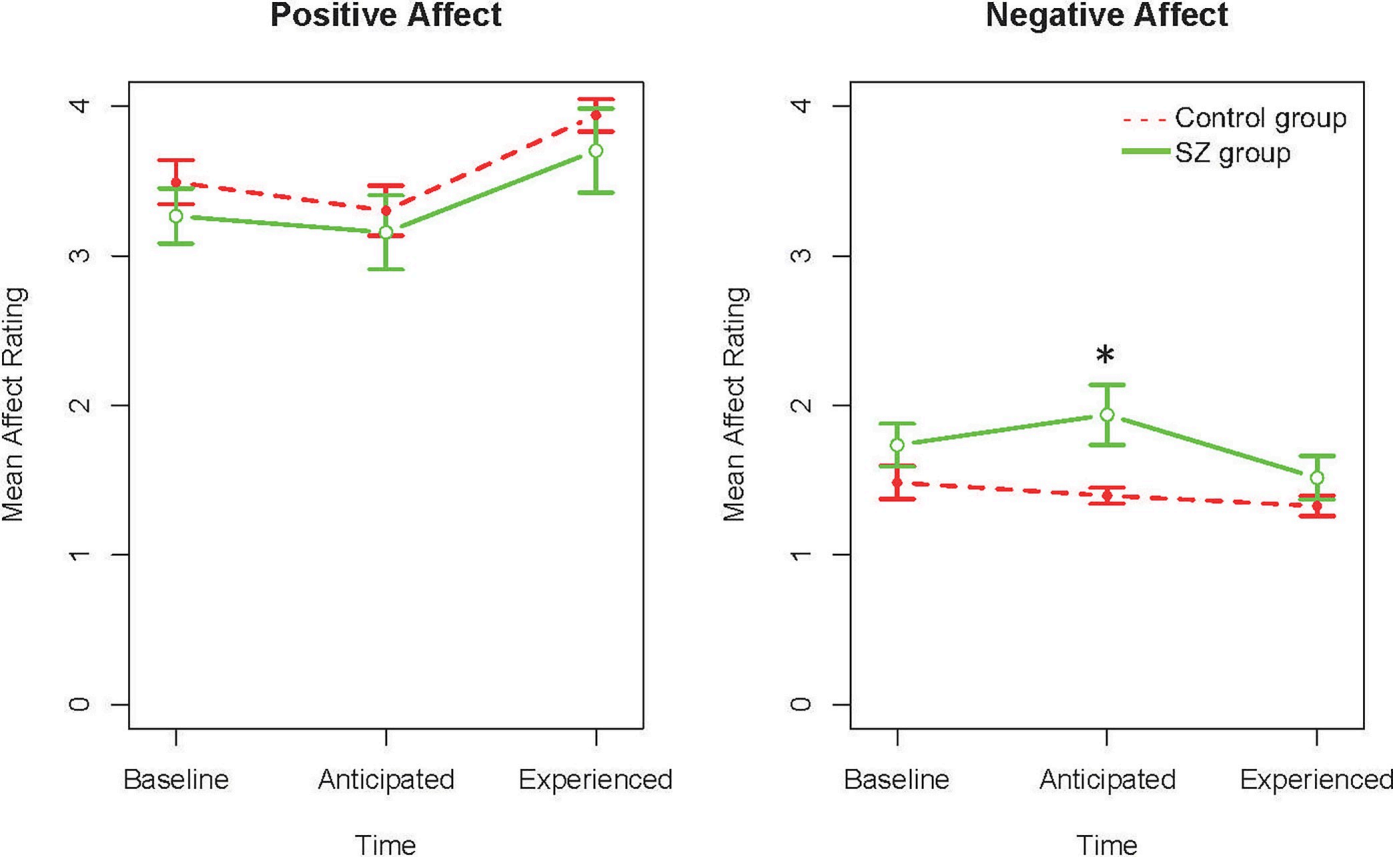
Positive and negative emotion ratings at each time point by group. The significant group difference is denoted with an asterisk.

We removed the non-significant 3-way interaction of Group, Emotion, and Time from the model (*F*(2, 226.85) = 0.23, *p* = .79), and reran the model. We found a significant Emotion X Time interaction, *F*(2, 227.1) = 9.57, *p* < .001, and a significant Emotion X Group interaction, *F*(1, 226.85) = 10.45, *p* = .001. As can be seen in Table 2 and Fig 1, although the groups did not differ in baseline levels of emotion or experienced emotion, the SZ group anticipated significantly more negative emotion than the Control group, *t*(43) = 3.29, *p* = .002, *d* = 1.0, 95% CI [-.06, 2.03]. There was no group difference in anticipated positive emotion, *t*(43) = 0.50, *p* = .62, *d* = .15, 95% CI[-.83, 1.13].

**Table 2:** Means (SD) of questionnaires measures by group.

	Schizophrenia (*n* = 16)	Control (*n* = 30)	Statistics
Baseline emotion			
Positive	3.27 (0.74)	3.49 (0.81)	*t*(44) = 0.93, *p* = .36, *d* = .28, 95% CI [-.71, 1.26]
Negative	1.73 (0.57)	1.48 (0.60)	*t*(44) = 1.37, *p* = .18, *d* = .41, 95% CI [-.59, 1.39]
Anticipated emotion			
Positive	3.16 (0.99)	3.30 (0.90)	*t*(43) = 0.50, *p* = .62, *d* = .15, 95% CI [-.83, 1.13]
Negative	1.94 (0.80)	1.40 (0.29)	*t*(43) = 3.29, *p* = .002, *d* = 1.0, 95% CI [-.06, 2.03]
Experienced emotion			
Positive	3.70 (1.13)	3.94 (0.58)	*t*(43) = 0.93, *p* = .36, *d* = .28, 95% CI [-.71, 1.26]
Negative	1.52 (0.58)	1.33 (0.37)	*t*(43) = 1.33, *p* = .19, *d* = .41, 95% CI [-.59, 1.39]
Interaction Evaluation			
Emotional Experience	25.69 (5.30)	28.47 (2.33)	*t*(44) = 2.48, *p* = .02, *d* = .75, 95% CI [-.28, 1.76]
Engagement	18.75 (5.14)	18.47 (3.46)	*t*(44) = 0.22, *p* = .83, *d* = .07, 95% CI [-.91, 1.05]
Disclosure	13.88 (2.24)	13.97 (1.75)	*t*(44) = 0.15, *p* = .88, *d* = .05, 95% CI [-.93, 1.03]
Quality	20.81 (4.02)	20.93 (2.80)	*t*(44) = 0.12, *p* = .91, *d* = .04, 95% CI [-.94, 1.02]
Affective Forecasting Accuracy			
Positive	0.55 (.75)	0.64 (.54)	*t*(43) = 0.47, *p* = .64, *d* = .14, 95% CI [-.84, 1.12]
Negative	-0.42 (.51)	-0.05 (.42)	*t*(43) = 2.64, *p* = .01, *d* = .81, 95% CI [-.23, 1.82]

In addition, both groups anticipated less positive emotion than they experienced (both *t*s > 2.90, both *p*s < .01) and also experienced an increase in positive emotion from baseline (both *t*s > 2.37, both *p*s < .05). At the same time, the SZ group anticipated more negative emotion than they experienced, *t*(15) = 3.30, *p* < .01, and there was a trend for them to experience a decrease in negative emotion from baseline, *t*(15) = 1.78, *p* = .095. There were no within-group differences for the Control group between baseline, anticipated, and experienced negative emotion, all *t*s < 1.1, all *p*s > .29. Last, we found that the SZ group was less accurate at forecasting negative emotion than the Control group, *d* = .81, 95% CI [-.23, 1.82], but there were no group differences in accuracy of forecasting positive emotion, *d* = .14, 95% CI [-.84, 1.12]. That is, the groups were equally inaccurate in their forecasts of positive emotion.

### Less positive emotional experience for the SZ group

Next, we tested whether the groups differed in their evaluation of the interaction. Although the groups did not differ in their ratings of the level of disclosure, engagement, or quality of the interaction (all *p*s > .82, all *d*s < .07), the SZ group reported significantly less positive emotional experience, *t*(44) = 2.48, *p* = .017, *d* = .75, 95% CI [-.28, 1.76]. This indicates that despite having similar evaluations regarding their partner’s amount of disclosure and level of engagement, as well as the overall quality of the interaction, the SZ group differed in their subjective emotional experience.

### Relationships between symptoms and measures related to the social interaction

As can be seen in Table 3, self-reported social anhedonia was positively correlated with anticipated negative emotion, *r* = .49, *p* = .001, and negatively associated with experienced positive emotion, *r* = -.59, *p* < .001. In addition, there was a negative association between self-reported social anhedonia and all subscales of the Interaction Evaluation measure, all *r*s *<* -.30, all *ps* < .05.

**Table 3:** Pearson Correlations among Emotion Ratings, Interaction Experience Ratings, and Symptom Ratings.

	1	2	3	4	5	6	7	8	9	10	11	12	13	14	15	16
1. Baseline PE	-															
2. Baseline NE	-.31*	-														
3. Anticipated PE	.76**	-.32*	-													
4. Anticipated NE	-.27	.52**	-.29	-												
5. Experienced PE	.69**	-.30*	.76**	-.35*	-											
6. Experienced NE	-.15	.39**	-.16	.59**	-.32*	-										
7. Eval-Emo Exper	.41**	-.25	.42**	-.32*	.72**	-.28	-									
8. Eval-Engagement	.26	-.33*	.35*	.04	.45**	-.06	.46**	-								
9. Eval-Disclosure	.44**	-.42**	.46**	-.20	.59**	-.48**	.47**	.39**	-							
10. Eval-Quality	.43**	-.36*	.41**	-.16	.62**	-.35*	.64**	.32*	.53**	-						
11. Social anhedonia	-.27	.48**	-.29	.49**	-.59**	.35*	-.69**	-.30*	-.37*	-.35*	-					
12. Reality Distortion	-.19	.24	-.08	.26	-.12	.25	-.23	-.003	-.17	-.24	.49**	-				
13. Disorganization	.14	.12	-.07	-.15	.29	.05	.28	.34*	.19	.17	-.04	.31*	-			
14. Diminish Express	-.13	.04	-.20	.23	-.36	.13	-.42**	-.04	-.02	-.02	.46**	.21	-.05	-		
15. Avolition-Apathy	-.18	.14	-.23	.40**	-.46**	.36*	-.44**	.01	-.13	-.16	.61**	.45**	.12	.72**	-	
16. CPZ equivalence	-.11	.25	-.19	.29	.05	.01	-.06	.17	-.05	-.03	-.02	.02	.27	-.22	-.34	-

Note: PE = positive emotion; NE = negative emotion; Eval-Emo Exper = Emotional Experience subscale of the Interaction Evaluation; Eval-Engagement = Engagement subscale of the Interaction Evaluation; Eval-Disclosure = Disclosure subscale of the Interaction Evaluation; Eval-Quality = Quality subscale of the Interaction Evaluation; Social Anhedonia = Revised Social Anhedonia Scale; Reality Distortion = psychotic symptom factor; Disorganization = disorganized symptom factor; Diminish Express = Diminished Expression/Inexpressivity symptom factor; Avolition-Apathy = asociality, avoltion, and anhedonia symptom factor

* p < .05

** p < .01

Similar to self-reported social anhedonia, the clinician-rated negative symptom factor of Avolition/Apathy was positively correlated with anticipated negative emotion, *r* = .40, *p* < .01, and was negatively correlated with experienced positive emotion, *r* = -.46, *p* < .01. The Avolition/Apathy factor was also negatively associated with the Emotional Experience subscale of the Interaction evaluation, *r* = -.44, *p* < .01. See S3 File for this study’s data.

## Discussion

Using the “36 questions that lead to love” [22] (i.e., Interpersonal Closeness Generation task) [13] to create a live social interaction, the current study assessed anticipated and experienced emotion in a SZ and a control group. Overall, results indicated that there were differences in predictions of future negative emotion and accuracy of these predictions between the groups and that these predictions were related to levels of self-reported social anhedonia. Self-reported social anhedonia was also associated with experienced positive emotion. These findings have implications for how social anhedonia is conceptualized as well as possible treatment targets.

First, although the groups did not differ in baseline levels of emotion, we found evidence that the SZ group anticipated significantly more negative emotion than controls but were less accurate in their prediction because they did not report experiencing the same amount of negative emotion as they thought they would. This is consistent with the findings of a daily diary study [21] that found people with SZ forecasted that they would experience more negative emotion over the course of a week than they actually reported experiencing. This is also consistent with self-reports of higher trait negative emotion in SZ than controls [35]. That is, people with SZ perceive themselves to have higher levels of negative emotion than controls, and consistent with this, they forecast that they will experience higher levels than controls in the future. Of note, given that participants made these predictions prior to meeting the confederate or having any information about the confederate, it is very unlikely that group differences in anticipated negative emotion are driven by some demographic characteristic of the confederate. Also, although the size of the effect of the difference between the groups was large in size (*d* = 1.0), the mean rating of anticipated negative affect for the schizophrenia group was below the middle of the scale (1.94 on a 5-point scale) but was still moderately correlated with self-reported social anhedonia (*r* = .49). This indicates that even a small increase in raw anticipated negative affect is related to increases in trait levels of social anhedonia.

One explanation for the increased, anticipated negative emotion reported in schizophrenia could be the accumulating effect of previous negative life experiences. Individuals with schizophrenia often endure early childhood adversity, stigmatization due to one’s psychological disorder, and marginalization due to their lower socio-economic status [36]. It stands to reason then that individuals with schizophrenia might have “learned” to expect frequent, negative life events, leading them to anticipate elevations in negative emotion in response to future occurrences, particularly in the face of uncertainty [37]. Thus, future research could test for the effect of uncertainty on emotions in schizophrenia as a possible mechanism of avolition-apathy negative symptoms.

At the same time, the groups did not differ in anticipation, experience, or accuracy in forecasting positive emotion. The finding that the groups were equally inaccurate in their prediction of positive emotion, taken together with evidence of the independence of positive and negative affect [38–41], suggests that there is not an anticipatory pleasure deficit associated with impending participation in a social activity described as “enjoyable” for our sample. Given that our SZ sample was relatively high functioning (e.g., stable outpatients, 44% employed at least part-time), it is possible that anticipatory pleasure deficits are limited to individuals who are lower functioning. Similar to the work of Strauss and Herbener [20], future research could aim to determine if there is a subgroup of individuals with schizophrenia with differential deficits related to the anticipation of positive emotion. Given that anticipatory pleasure deficits are theoretically linked to motivation to engage in future social events, identifying a group of people with this deficit could be a way to individualize treatment targets, a goal of NIH’s Precision Medicine Initiative [42], and increase social motivation.

We also found no group difference in experienced positive emotion. This finding is consistent with reviews that report experiences of positive emotion in response to evocative stimuli to be similar between SZ and control groups [1,3,43]. Thus, consistent with these reviews, we found that consummatory pleasure in SZ appears intact. At the same time, we found that decreased experienced positive emotion was associated with self-reported social anhedonia and clinician-rated Anhedonia-Asociality. This is consistent with previous research that has reported that Anhedonia–Asociality scores from the SANS were negatively associated with self-reported emotions in response to pleasant films clips [5]. Overall, this indicates that rather than being a global deficit associated with SZ group as a whole, pleasure deficits may be specific to anhedonia.

Despite a lack of differences in predicted or experienced positive emotion or differences in ratings of the overall quality, level of engagement, or amount of disclosure, controls reported finding the interaction to be a more positive emotional experience overall. This effect was medium-to-large in size (*d* = 0.75, 95% CI [-.28, 1.76]) and was correlated with self-reported social anhedonia. This suggests that other factors may be driving the group difference in overall emotional experience, and one possible factor is low-pleasure beliefs. Low-pleasure beliefs have been associated with anhedonia [44], and it has been argued that disrupted memory processes (e.g., encoding, retrieval) [9] and elaborative processing abilities [45] may perpetuate such beliefs. That is, despite contrary evidence (e.g., the experience of increased positive emotion in response to a recent, pleasant social interaction), individuals with anhedonia may report a social interaction was less positive overall compared to controls because they do not believe they enjoy social interactions. Thus, another potential treatment target would be to focus on such dysfunctional beliefs. For example, using a cognitive-behavioral approach that incorporates experiential social encounters, such as through virtual reality, might be an effective way to modifying these beliefs because of the live, directed feedback that is possible.

Of note, both self-reported social anhedonia and Avolition-Apathy symptoms were moderately correlated with Reality Distortion symptoms. Thus, it is possible that these symptoms are secondary to psychotic symptoms. If so, the most effective treatment for such symptoms would be different [46,47]. Future research should consider this possibility when addressing social abnormalities.

Last, although the effects of group differences found were large, the sample size of the current study was relatively small and mean negative emotion ratings were relatively low. Thus, replication of the current findings is needed. However, using a novel approach that minimized the reliance of higher-order cognitive processes, this study was a first step toward overcoming some problems with previous research on affective forecasting in SZ. Overall, we found that self-reported social anhedonia was associated with predictions of negative emotion and experiences of positive emotion as well as evaluations of the social interaction. This indicates that anhedonia may involve a dynamic interplay between positive and negative emotion, which may impact one’s evaluation of a social interaction.

## Supporting information

S1 File Appendix. Cover story provided to participants in the social interaction task.
(DOCX)

S2 File Appendix. Social Interaction Task questions and confederate responses.
(DOCX)

S3 File Data. Study data.
(CSV)
