# Supplementary material for: Emotional response in schizophrenia to the “36 questions that lead to love”: Predicted and experienced emotions regarding a live social interaction
Source: PLoS One. 2019 Feb 27;14(2):e0212069. doi: 10.1371/journal.pone.0212069 (PMC6392255; doi:10.1371/journal.pone.0212069)
Supplement: S1 File — Cover story provided to participants in the social interaction task. (DOCX) [file pone.0212069.s001.docx]

Cover Story for the Social Interaction Task

The following was read to every participant at the start of the Social Interaction Task:

“This next task is a sharing game. You will have a partner, who is also a participant in this study, and the game involves asking and answering questions with your partner. In a few moments your partner will be joining us here in this room. She is enrolled in this same study and has been going through the same procedures you have. Your task, which we think will be quite enjoyable, is simply to get close to your partner. We believe the best way for you to get close to your partner is for you to share with them and for them to share with you.

When she comes over, you will sit together and read through a binder with a number of questions. These questions are to help you get to know your partner a little better. You and your partner will receive more detailed instructions when you are together.”
