# Supplementary material for: Emotional response in schizophrenia to the “36 questions that lead to love”: Predicted and experienced emotions regarding a live social interaction
Source: PLoS One. 2019 Feb 27;14(2):e0212069. doi: 10.1371/journal.pone.0212069 (PMC6392255; doi:10.1371/journal.pone.0212069)
Supplement: S2 File — Social Interaction Task questions and confederate responses. (DOCX) [file pone.0212069.s002.docx]

1) Given the choice of anyone in the world, whom would you want as a dinner guest?

I think I would choose Albert Einstein. He is a such a physics icon, known by pretty much everyone even though his research was pretty sophisticated. That is incredible. I really admire the way he saw the universe differently from other people, and opened up a whole new way of thinking of things. How often does that happen in history? Not often! Not only was he brilliant, but he had humble beginnings. I think he would make a fascinating dinner guest.

2) Would you like to be famous? In what way?

I can’t say I would like to be famous… but I guess I would like to be well-known. Only for things I have worked for though. In my career, I like to be well-known as an actuary, because people regard me as an expert and come to me with questions. Being well-known opens up a lot of opportunities that way. But I would hate to be famous, people following you around and digging into your personal life? Definitely not for me!

3) Before making a telephone call, do you ever rehearse what you are going to say? Why?

Yeah, well, I used to do this a lot. Probably more when I was younger than now but still do it now. I still do it now when I'm calling someone I don't know or calling someone to ask for something. I think I feel less anxious when I practice or even write down what I'm going to say before calling.

4) What would constitute a "perfect" day for you?

I think it would start with me being able to sleep in as late as I want. Too often now I have to get up at a certain time for something, even on the weekends. Then, I would relax in bed with iced coffee and chocolate covered almonds, reading the news or a good book for a while. Then I would spend some time outdoors in the sunshine, maybe a long walk at the beach, and probably sit outside and read something interesting. Then I would get ready at home and head out for some champagne and a fancy dinner at a foodie spot somewhere around, probably in Newport. Lots of relaxing, time in nature, learning something, and ending with champagne—that would be a perfect day!

5) When did you last sing to yourself? To someone else?

I can’t even remember the last time I last sang to myself! I am definitely not a big singer on my own! I did adopt a cat a couple of weeks ago though. Sometimes I sing him a lullaby from my own childhood to relax him. He seems to like it!

6) If you were able to live to the age of 90 and retain either the mind or body of a 30-year-old for the last 60 years of your life, which would you want?

Definitely retain the mind of the 30 year old. I'm pretty scared about losing "it". What would be the point of looking good if I didn't have it all together up here? Ha, but that's just my opinion!

7) Do you have a secret hunch about how you will die?

I don't think I have thought about it much…I guess I think that I'll probably die of a stroke in my sleep when I'm in my 80s or 90s like my grandpa did. Not sure if that’s just wishful thinking or not, but that would be a great way to go, wouldn’t it? A nice long life and then just a peaceful end.

8) Name three things you and your partner appear to have in common.

SHOULD BE TRUE BASED ON WHAT HAS BEEN GOING ON SO FAR.

9) For what in your life do you feel most grateful?

I am most grateful for my family. I am very lucky to have people that I can ask for anything anytime. This doesn't mean that they all would do anything but I know that at least one would say, "yes", if I really needed something.

10) If you could change anything about the way you were raised, what would it be?

I think I would change how stressed out my parents were about work when I was younger. They both worked really hard and were always stressed about something happening at work or whether their job was secure. I don't think they wanted to stress us kids out, but we heard about it a lot—at least it felt like that at the time—and it definitely made me worried a lot.

11) If a crystal ball could tell you the truth about yourself, your life, the future, or anything else, what would you want to know?

Maybe it's because we were just talking about this, but I would probably want to know when I am going to die. If I knew the exact date it was going to happen, then I could plan to make sure I do everything I want to before that date. Kinda morbid but I think it would be helpful to prevent any feelings of regret. I would have no excuses of not doing things because I'd know exactly how much time I have left.

12) Is there something that you've dreamed of doing for a long time? Why haven't you done it?

I have dreamed of going into space for a long time. When I was little I thought I wanted to be an astronaut. I haven’t done it yet because I did not actually become an astronaut! But now, I hope to go on a commercial space flight some day!

13) What is the greatest accomplishment of your life?

This would have to be earning my Fellowship in the Society of Actuaries. I worked so, so hard for it and am very proud of it. It takes an average of 10 years after getting your math degree to accomplish this, and most people drop out or stop partway through at a lesser designation! I still feel pride when I am asked about it or have to tell someone about it. It was where I really learned to not just rely on natural intellect or math skills coming easily, but to discipline my mind and to stick with something that was really, really challenging and even at times discouraging. Most of my friends dropped out!

14) What do you value most in a friendship?

I most value "reliability" in a friend. Like you know the person will call you back when you call, be where they saw they will be when they say they will be there, keeps promises, that kind of thing. Basically, someone who sticks to their word. A friend who can't do that isn't really much of a friend!

15) What is one of your most treasured memories?

One of my best memories is the trip I took to Iceland last year. I had heard it was a life-changing place, and I decided to go there by myself! It was an absolutely amazing experience from start to finish. The landscape was so surreal, it constantly felt like being in another world. It kind of made you feel like you were connected to something bigger, or seeing something bigger for the first time. I don’t know, it was just amazing. The people were so friendly, and there were all these cool places to explore, enormous waterfalls, black sand beaches, just incredible nature everywhere. That trip was a memory I will always treasure!

16) What is one of your most terrible memories?

One of my most terrible memories is getting into a car accident with my best friend back in college. She was driving, and we were in Seattle (where I’m from), and it was raining so hard that it was hard to see the road. She got confused about which exit we needed to take, and all of the sudden out of nowhere she seemed to think we were supposed to take the exit that we were almost passed! (It wasn’t even the right exit!) So out of the blue, she jerked the wheel really hard to try to make the exit. There was no way we could have made it, but especially with the rain, the car spun out and we crashed into a bunch of water barrels that were protecting the concrete divider. It was one of the scariest moments! Luckily we didn’t hit anyone else. But we both had injuries, and I ended up having pretty bad pain for the rest of that school year, which made it really hard to focus. It was just a terrible event.

17) If you knew that in one year you would die suddenly, would you change anything about the way you are now living? Why?

If I really had only one year left, I would probably immediately start traveling full time! There are so many places I still want to see.

18) What does friendship mean to you?

Friendship means being able to count on each other—kinda what I was talking about earlier. Also, having good times together—laughing, sharing stories, probably gossiping too—but always sharing bad times—when things aren't going well, being able to share that with each other too. True friends share both good and bad things. People who you only share good things with are more like acquaintances to me.

19) What roles do love and affection play in your life?

Both of these are really important to me. My family and I are really affectionate. I don't feel like I need to be loved by or love lots of people, but at least one or two is really important…Yeah, I would say they are both really important to me but not something I think about a lot...probably because I think I have both!

20) How close and warm is your family? Do you feel your childhood was happier than most other people's?

My two sisters and I are very close! We are in touch almost every day, and spend all our birthdays and holidays together no matter what else is going on in our lives! I don’t think my childhood was happier than most, but I’m so happy to have such a meaningful relationship with my sisters, it’s one of the most important aspects of my life!

21) How do you feel about your relationship with your mother?

Overall, I think it is pretty decent now. We didn't get along very well when I was younger. I was a good kid, not doing bad things, no detentions or things like that, but she was still always suspicious I would get into trouble. I think she probably read some parenting book about teenagers! Hah! We get along much better now that I am grown up.

22) Alternate sharing something you consider a positive characteristic of your partner. Share a total of five items.

SHOULD BE TRUE BASED ON WHAT HAS BEEN GOING ON SO FAR.

23) If you were going to become close friends with a new person, what would be important for him or her to know?

I guess it would be important for a person to know that if I say I am going to do something, I will do it, and I expect the other person to do the same. It goes back to that "reliable" quality that I think is really important.

24) Tell your partner what you like about him/her. Be very honest this time saying things that you might not say to someone you've just met.

SHOULD BE TRUE BASED ON WHAT HAS BEEN GOING ON SO FAR.

25) Share with your partner an embarrassing moment in your life.

I had to talk in front of about 400 people and was very nervous about it. I got dressed up and debated about what shoes to wear. I went against my better judgment and wore shoes that weren't comfortable and were a little too high because I thought they looked more professional. Anyway, I was giving the talk and I thought it was going pretty well. I walked around a bit and as I was walked back towards where I started, my heel got caught on a snag on the rug and I tripped. I was so embarrassed. There as a mixture of quiet giggles and gasps of concern from the audience. It was horrible! I made a joke that I should haven't worn those shoes and just kept going. I was so happy when it was over. I have never worn those shoes again!

26) When did you last cry in front of another person? By yourself?

This is kind of weird, but the last time I cried in front of another person was when I saw the movie Arrival at the movie theater! I’m not sure if you’ve seen that movie, it’s the one about aliens who perceive time in a different way from us, and they teach Amy Adams’ character to see it that way too, by teaching her their language! It’s such an interesting idea, and I’ve always been interested by the nature of time. Anyway it wasn’t even really a sad movie, but at the end she is able to see into her future and she sees some tragedy but also sees that she chooses the same life anyway. I totally started crying at the end of the movie, and had to wait until the credits finished to leave! I was kind of embarrassed! I don’t remember the last time I cried by myself, probably a sad movie. Sad movies get me every time!

27) What, if anything, is too serious to be joked about?

Wow. I guess I would say war is something that is too serious to be joked about. I know some famous comedians sometimes get into the whole political joke realm, but I think joking about war is going too far, given the fact that real people risk their lives or die in these scenarios.

28) If you were to die this evening with no opportunity to communicate with anyone, what would you most regret not having told someone? Why haven't you told them yet?

I think I would most regret not telling my family how much they mean to me and how much I love them. I do let them know pretty often that I love them, but if I knew this was the end, I would wish I could let them know one more time!

29) Your house, containing everything you own, catches fire. After saving your loved ones and pets, you have time to safely make a final dash to save any one item. What would it be? Why?

Honestly, I try not to stay too attached to any of my physical belongings. But I would be totally lost without my laptop. It contains not only pictures from my experiences, but papers, essays, journals, everything! I would definitely go back in to grab it.

30) Share a personal problem and ask your partner's advice on how he or she might handle it. Also ask your partner to reflect back to you how you seem to be feeling about the problem you have chosen.

Something weird recently happened with a co-worker. I had always thought we got along just fine—we are polite to each other and had small talk once in a while. We had hung out in a group before and had never had any kind of fight or disagreement at least that I'm aware of. Anyway, recently, a group of us were standing in the hallway talking before a meeting and she started to tell a story about something that happened at a party she had just had. Based on others' reactions and comments, it was pretty clear that they all had been invited and had gone. Well, I hadn't been invited and this was the first I had heard about it. It was really awkward for me, and I think at least one other person realized what was going on because she tried to change the topic. I just don't understand why I wasn't invited and how to interact with her now.

YOUR RESPONSE TO THEM SHOULD BE LIVE/GENUINE.
